# Cross-sectional evaluation of clinical and immunological parameters at partially microgrooved vs machined abutments in humans

**DOI:** 10.1186/s40729-021-00329-8

**Published:** 2021-05-25

**Authors:** Gerhard Iglhaut, Sebastian Salomon, Tobias Fretwurst, Peter Thomas, Janina Endres, Selina Kessler, Burkhard Summer

**Affiliations:** 1 Department of Oral and Craniomaxillofacial Surgery, Translational Implantology, Center for Dental Medicine, University Medical Center of Freiburg, Freiburg, Germany; 2 Private Center of Oral Surgery, Bahnhofstrasse 20, 87700 Memmingen, Germany; 3 Private Dental Center, Frastanz, Austria; 4 Department of Dermatology and Allergology, Ludwig Maximilians University of Munich, Munich, Germany

**Keywords:** Laser-microtextured surface abutments, Machined abutments, Peri-implant crestal bone loss, Peri-implant crevicular fluid (PICF), Cytokines IL-1α, IL-1β, IL-6, IL-8, and IL-10

## Abstract

**Objective:**

The objective of the present study was to examine the clinical and immunological parameters in samples collected from the peri-implant crevicular fluid (PICF) of machined titanium (M) abutments compared to titanium abutments with a laser-microtextured surface (LMS) on dental implants.

**Material and methods:**

A total of 40 patients with one titanium implant, half of them (*n*=20) provided with a M abutment (control group) and the other half (*n*=20) with LMS abutments (test group), were included in the study. Clinical parameters pocket probing depth (PD), full-mouth plaque score (FMPS), radiographic bone loss (RBL), clinical attachment level (CAL), mucosal recession (MR), bleeding on probing (BOP), and width of keratinized mucosa (KM) were evaluated. The peri-implant sulcus fluid was analyzed for cytokines IL-1α, IL-1β, IL-6, IL-8, and IL-10 via flow cytometry.

**Results:**

Clinical evaluation demonstrated no significant difference of PD (mean LMS = 3.50 mm/SD 0.95 mm vs mean M = 3.45 mm/SD 0.76 mm (*p*=0.855)), MR (mean LMS = 0.30 mm/SD 0.57 mm vs mean M = 0.35 mm/SD 0.67 mm (*p*=0.801)), CAL (mean LMS = 3.60 mm/SD 1.14 mm vs mean M = 3.55 mm/SD 0.89 mm (*p*=0.878)), and KM (mean LMS = 2.03 mm/SD 1.08 mm vs mean M = 2.13 mm/SD 0.92 mm (*p*=0.754)) between LMS and M abutments. LMS abutments showed less BOP than M abutments (26.7% vs 30.8%), but statistically not significant (*p* = 0.2235). Radiographic bone loss (mean LMS = 0.22 mm/SD 0.44 mm vs mean M = 0.59 mm/SD 0.49 mm) was reduced in the test group in comparison with the control group (*p*=0.016). In the collected PICF, the levels of pro-inflammatory cytokines IL-1α (median LMS = 180.8 pg/ml vs M = 200.9 pg/ml (*p*=0.968)) and IL-1β (median LMS = 60.43 pg/ml vs M = 83.11 pg/ml (*p*=0.4777)) were lower, and the levels of IL-6 (median LMS = 180.8 pg/ml vs M = 200.9 pg/ml (*p*<0.0001)) were significantly lower in the test group. In contrast, the levels of IL-8 (median LMS = 255*.*7 pg/ml vs M = 178*.*7 pg/ml (*p*=0.3306)) were higher in the test group, though not significantly. The levels of anti-inflammatory IL-10 were significantly increased in the test group (LMS median = 0.555 pg/ml vs M median = 0.465 pg/ml (*p*=0.0365)). IL-1β showed a significant correlation to radiologic bone loss (*p*=0.0024). The other variables IL-1α, IL-6, IL-8, and IL-10 had no significant correlation to radiological bone loss.

**Conclusion:**

Within the limitations of this study, titanium implants provided with laser-microtextured surface abutments seem to demonstrate less pro-inflammatory and more anti-inflammatory activity and to show reduced radiographic bone loss compared to machined titanium abutments.

**Clinical relevance:**

The use of laser-microtextured surface abutments might have the potential to support peri-implant tissue health.

## Introduction

Endosseous dental implants are utilized to replace missing teeth or to support full- or partial-arch prostheses [1, 2]. Wound healing, osseointegration, and tissue stability to dental implants and abutments may depend on alloy composition, surface chemistry and texture, implant design, abutment connection and design, and additional repeated removal of abutments [3–5]. The peri-implant soft tissue attachment is seen as a biological seal from the highly contaminated oral environment to prevent peri-implant hard and soft tissue infection [6]. Implants affected by peri-implantitis are characterized by marginal bone loss, bleeding on probing, and eventually peri-implant pockets or implant loosening [7, 8]. Therefore, the clinical diagnostic criteria include bleeding on probing, probing depth, and mobility in combination with radiographic examination [7]. However, these diagnostic methods may possibly not be specific and sensitive enough to differentiate the early onset and progression of peri-implantitis, since probing is influenced by the direction and the force used by the examiner [9, 10]. Hence, additional diagnostic tools including possible biomarkers out of the peri-implant crevicular fluid (PICF) might have a potential for monitoring the early and late stages of peri-implant pathologies but needs to be proven in the future [8, 11]. The onset of peri-implantitis is initiated by the host response to bacterial plaque biofilm formation and associated with clinical signs of inflammation [12–14]. Further, it has become apparent that the material could have an influence [15]. Cytokines, chemokines, and other mediators induce osteoclast activity and the upregulation of further pro-inflammatory enzymes [16]. The most investigated pro-inflammatory cytokines in PICF include IL-1β, IL-6, IL-8, and TNF-α followed by anti-inflammatory cytokines IL-4 and IL-10 [17]. Increased IL-1β levels were first described in experimental gingivitis studies at the teeth [18]. The pro-inflammatory enzymes are involved in the degradation of extracellular matrix proteins like laminin, collagens, or fibronectin leading to an increase in inflammatory cell migration and further tissue destruction [17, 19].

To prevent peri-implant inflammation, recent studies focused on a distinctive laser-generated microgrooved surface (LMS) in the cervical region of implants and titanium abutments. These surfaces might seal the implant against infections by building a strong connective tissue adherence and could therefore show less inflammatory activity and consequently a decrease in marginal bone loss in comparison with other abutment surfaces [20–23]. LMS surface is a highly orientated microgeometry produced by using a computer-controlled laser system. In vitro studies demonstrated a direct fibroblast and osteoblast attachment to LMS [24, 25]. Nevins et al. confirmed these findings later histologically in animal and human models [26, 27]. Trabecular bone adjacent to LMS grows strongly parallel to the microgrooves [28]. The soft tissue attachment on LMS implants is reported to be much different from the traditional attachment providing fibers orientated parallel and circumferential to the implant collar surface. Fibers on LMS seem to be orientated perpendicularly to the surface similar to the teeth and demonstrate functionally orientated collagen fibers mechanically attached to the microgrooves [27, 29].

The aim of this cross-sectional, retrospective study was to analyze whether the surface characteristics of LMS abutments compared to M abutments influence the clinical parameters and if clinical parameters are correlated with inflammatory activity assessed by measuring cytokines in the PICF. The hypothesis of the present study is, based on previous clinical studies [14, 30], that LMS abutments might induce lower levels of pro-inflammatory and higher levels of anti-inflammatory cytokines in the peri-implant crevicular fluid and consequently different peri-implant bone loss compared to conventional machined abutments.

## Material and method

### Study design

The two-center study was designed as a retrospective, cross-sectional observational study. The study was conducted in accordance with the requirements of the 1964 Helsinki Declaration. Ethical approval was approved by the ethics committee of the Ludwig Maximilian University of Munich (Project no. 17-132/28.03.2017). Before enrollment, the patients received information regarding the purpose of the study and signed an informed consent. Forty patients were included in the study, generally healthy adult females and males between the age of 18 and 80 years with one standard titanium dental implant in the posterior maxilla or mandible provided with a single full ceramic monolith crown minimum 2 up to 5 years after insertion. Patient selection (each center *n*=20) followed in row related to a hygiene appointment during a time period of 8 weeks in July/August 2018 at two private dental centers in Memmingen, Germany, and in Frastanz, Austria.

**Fig. 1:**
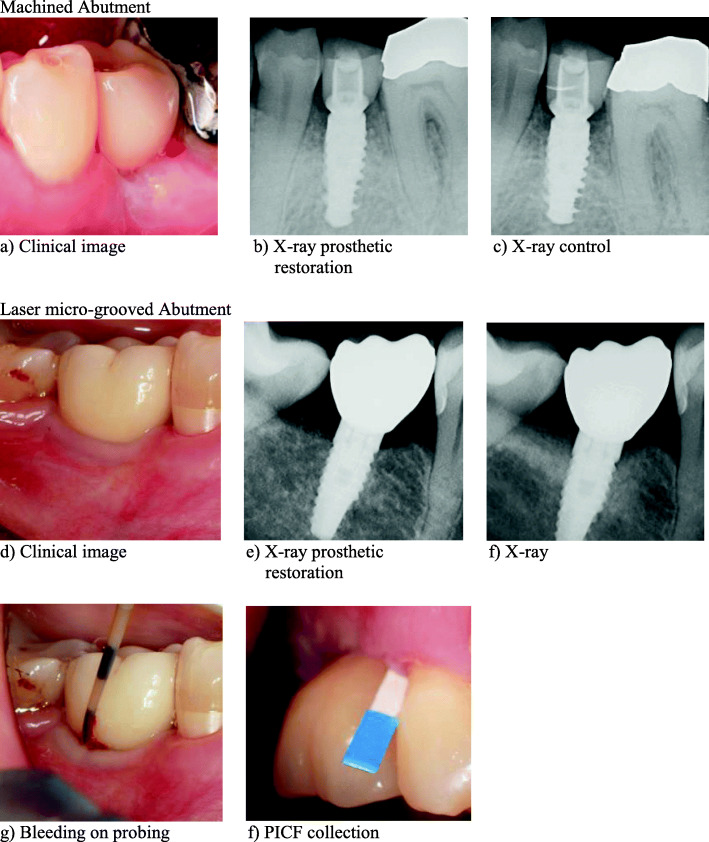
Clinical and radiographic images. Machined abutment: **a** clinical image, **b** X-ray prosthetic, and **c** X-ray control restoration. Laser microgrooved abutment: **d** clinical image, **e** X-ray prosthetic, **f** X-ray restoration, **g** bleeding on probing, and **f** PICF collection

The test group (*n*=20) provided with LMS abutments (Simple Solution, BioHorizons, Birmingham, USA) on Laser-Lok Tapered Internal Implants (BioHorizons, Birmingham, Alabama, USA) was evaluated at the center Memmingen while the control group (*n*=20) with M abutments (Variobase, Straumann, Basel, Switzerland) on Straumann Bone Level (Straumann, Basel, Switzerland) at the center Frastanz. Study data were analyzed retrospectively. Clinical, immunological, and radiographic parameters of every proband were assessed cross-sectionally during the hygiene appointment (Fig. 1).

### Inclusion criteria

The following are the inclusion criteria:

1. Subjects must have a voluntarily signed the informed consent form before any study-related action.
2. Males and females with an age of 18 up to 80 years of age.
3. Non-smokers and smokers < 20 cigarettes/day.
4. Titanium implants in the posterior maxilla and mandible minimum 2 up to 5 years after prosthetic loading.
5. Full-mouth plaque must be ≤ 25% at the time of hygiene appointment.

### Exclusion criteria

The following are the exclusion criteria:

1. Systemic disease that would interfere with dental implant therapy (e.g., uncontrolled diabetes)
2. Mucosal diseases (e.g., erosive lichen planus)
3. History of local irradiation therapy or malignancies
4. Current untreated periodontitis or gingivitis
5. Any untreated endodontic lesions
6. Severe bruxing or clenching habits
7. Patients with inadequate oral hygiene or unmotivated for adequate home care
8. Conditions or circumstances, in the opinion of the investigator, which would prevent completion of study participation or interfere with the analysis of study results, such as a history of non-compliance or unreliability
9. Physical or mental handicaps that would interfere with the ability to perform adequate oral hygiene
10. Pregnant or breastfeeding women
11. Patients on anti-inflammatory medication

### Clinical and radiographic investigation

**Fig. 2:**
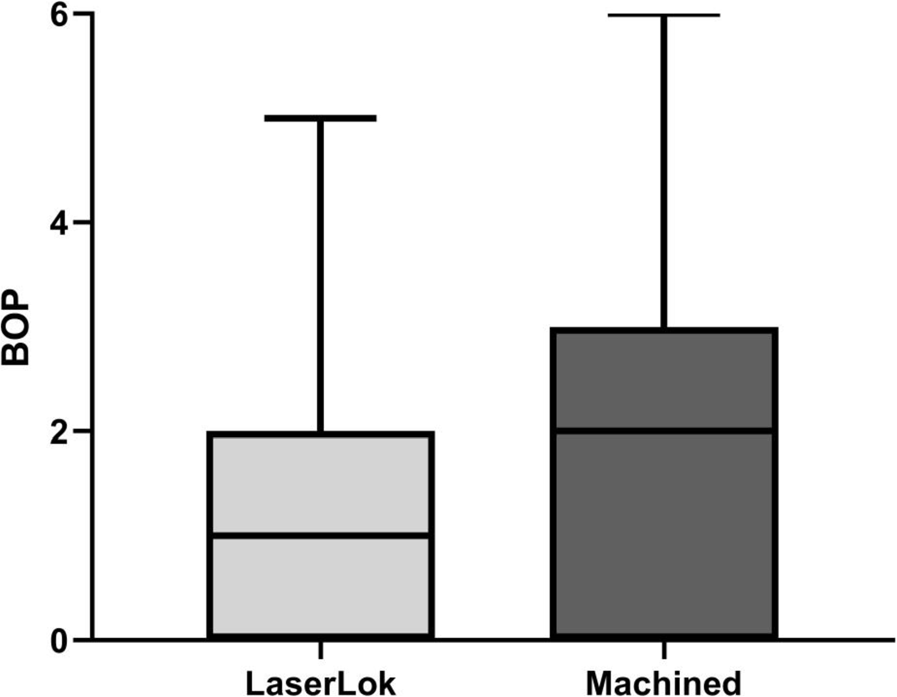
Bleeding on probing in numbers

All clinical measurements (Fig. 2a, d) were performed by two calibrated dentists during a single hygiene appointment of every patient using a PCP-12 Parodontal Probe (HuFriedy, Chicago, IL, USA) (Fig. 2g). The clinical parameters recorded were full-mouth plaque score (FMPS), bleeding on probing (BOP), probing pocket depths (PD), clinical attachment level (CAL), width of fixed mucosa (FM), and mucosal recession (MR). The radiological examination was performed during the same appointment as part of the routine follow-up (Fig. 2c, f). These digital radiographic images (parallel technique and measurements) were compared to the radiographic image taken immediately after the implant surgery procedure using the Byzz Ray Software (Orangedental, Biberach, Germany) (Fig. 2b, e). Measurements were taken mesial and distal interproximal. The statistical analysis was made with the mean value of the two variables taken.

### Inter-rater calibration

The inter-rater calibration for PD was performed by the two investigators in 10 probands on 2 teeth and 2 implants each on 6 sites (mesio-buccal, mid-buccal, disto-buccal, mesio-lingual, mid-lingual, and disto-lingual). The statistical analysis resulted in a kappa of 0.773 and an agreement of 86.04% (Table 1).

**Table 1:** Inter-rater calibration

Agreement	Expected agreement	Kappa	Std. Err.	*Z*	Prob>*Z*
86.04%	47.73%	0.7330	0.0392	18.69	0.0000

### PICF sample collection

After gentle air-drying and isolation with paper rolls, peri-implant sulcus fluid samples were obtained at five aspects (mesio-buccal, mid-buccal, disto-buccal, mesio-lingual, disto-linguall) of the target implant site with sterile paper strips (Periopaper, Oraflow Inc., Hewlett, NY, USA). The strips were placed in a sulcus depth of 1–2 mm (Fig. 2f) and were remained 30 s in the sulcus in accordance with previously published procedures [14, 30]. Paper strips contaminated with blood were not used for examination. Subsequently, the strips were collected in cryotubes, transported in ice, and stored in a refrigerator at − 80°C.

### Analysis of cytokines by cytometric bead assay (CBA)

Simultaneous analysis of IL-1α, IL-1β, IL-6, IL-8, and IL-10 was performed by flow cytometry via cytometric beads assay (CBA) in the laboratory AllergoMat (Clinic and Policlinic for Dermatology and Allergology, Ludwig Maximilian University, Munich, Germany) according to a previously published protocol [31]. The eppan tubes with the paper strips were defrosted and saturated with 300 μl PBS. Thirty minutes later, 300 μl fluid was obtained and again frosted in eppan tubes. For the CBA (BD Biosciences, Heidelberg, Germany), a standard series with ten tubes with increasing dilution each with 500 μl fluid was produced. The dilution standard series was started with 4 ml assay diluent for the top standard and produced with nine other tubes with each 500 μl assay diluent. The analysis of the cytokines was then performed via flow cytometry (BD FACS Canto, BD Biosciences, San Jose, CA, USA). Concentrations were given as pg/ml.

### Statistical method

The power calculation (default 0.80) related to IL-1beta resulted in a sample size of 19 in each group. Statistical analysis was performed with IBM SPSS Statistics (SPSS version 23.0, IBM, Armonk, NY, USA). The mean, median values, and standard deviations were calculated for each variable and group. The unpaired *t* test and Mann-Whitney *U* test were used at a significance level *p*<0.05. Sample size calculation was not performed due to the fact of missing prior reference studies.

## Results

### Patient cohort

The mean age of the study cohort was 61.48 years (M mean age = 61.25 years, LMS mean age = 61.70 years). The control group contained 13 male and 7 female patients, while in the test group, 13 patients were female and 7 patients male (Table 2). The mean loading time of M abutments was 2.5 up to 3.5 years (mean 2.6 years, SD 0.66) and of LMS abutments 2.5 up to 4.25 years (mean 3.6 years, standard deviation SD 1.27) (Table 2). In both groups, only one smoker was included. Hence, it was not considered in the statistics.

**Table 2:** Patient data

Implant type	Abutment type	Number	Mean age	SD	Male	Female	Loading time (years)	SD
Tapered internal	LMS	20	61.70	11,662	7	13	3.6	1.27
Bone level	Machined	20	61.25	13,266	13	7	2.6	0.66
Total		40	61.48		20	20		

### Clinical parameters

Clinical evaluation demonstrated no significant difference in PD (mean LMS = 3.50 mm/SD 0.95 mm vs mean M = 3.45 mm/SD 0.76 mm (*p*=0.855)), MR (mean LMS = 0.30 mm/SD 0.57 mm vs mean M = 0.35 mm/SD 0.67 mm (*p*=0.801)), CAL (mean LMS = 3.60 mm/SD 1.14 mm vs mean M = 3.55 mm/SD 0.89 mm (*p*=0.878)), and KM (mean LMS = 2.03 mm/SD 1.08 mm vs mean M = 2.13 mm/SD 0.92 mm (*p*=0.754)) between LMS and M abutments (Tables 1 and 3). LMS abutments showed less BOP than machined abutments (26.7%/mean numbers 1.32/SD 1.282 vs 30.8%/mean numbers 1.85/SD 1.599, respectively) as shown in Fig. 2, but statistically not significant (*p*= 0.2235).

**Table 3:** Clinical parameters PPD, MR, CAL, BOP, KM, and RBL for M vs LMS abutments

Clinical parameters	Abutment type	Mean mm	SD	*p* value
PPD	LMS	3.50	0.95	0.855
	Machined	3.45	0.76	
MR	LMS	0.30	0.57	0.801
	Machined	0.35	0.67	
CAL	LMS	3.60	1.14	0.878
	Machined	3.55	0.89	
KM	LMS	2.03	1.08	0.754
	Machined	2.13	0.92	
RBL	LMS	0.22	0.44	0.016
	Machined	0.59	0.49	

Routine follow-up radiographic examinations were compared to the initial radiographic images at the time of implant placement. The radiographic bone loss was significantly higher (*p*= 0.016) in the control group (mean M = 0.59 mm; SD = 0.49) than in the test group (mean LMS = 0.22 mm; SD = 0.44) as represented in Table 2.

### Immunological analysis

PICF samples obtained from patients in the test group had significantly lower pro-inflammatory IL-6 levels (median LMS = 180.8 pg/ml vs M = 200.9 pg/ml (*p*<0.0001)) as presented in Table 4 and Fig. 4. The levels of cytokines IL-1α (median LMS = 180.8 pg/ml vs M = 200.9 pg/ml (*p*=0.968)) and IL-1β (median LMS = 60.43 pg/ml vs M = 83.11 pg/ml (*p*=0.4777)) were lower in the study group, but statistically not significant. In contrast, the levels of IL-8 (median LMS = 255*.*7 pg/ml vs M *=* 178*.*7 pg/ml (*p*=0.3306)) were higher in the test group, though not significantly. The anti-inflammatory IL-10 was significantly increased (*p*=0.0365) in the test group (median = 0.555 pg/ml) compared to the control group (median = 0.465 pg/ml)*.*

**Table 4:** Cytokine median level for LMS vs M abutments

	IL-1a	IL-1b	IL-6	IL-8	IL-10
LMS median	180.8	60.43	0.250	255.7	0.5550
Machined median	200.9	83.11	0.920	178.7	0.4650
*p* value	0.968	0.4777	<0.0001	0.3306	0.0365

**Fig. 3:**
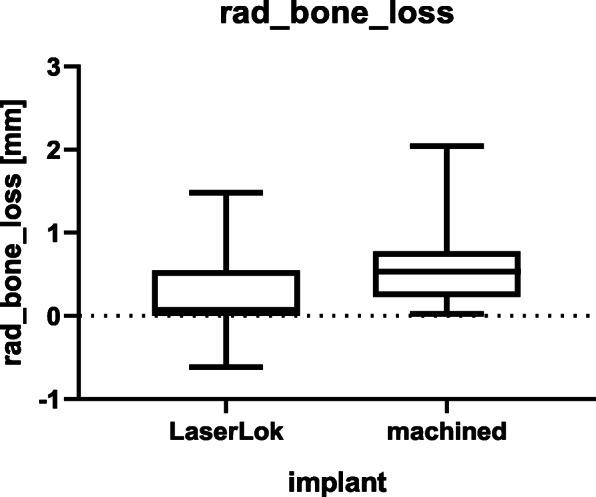
Radiologic bone loss in millimeters

**Fig. 4:**
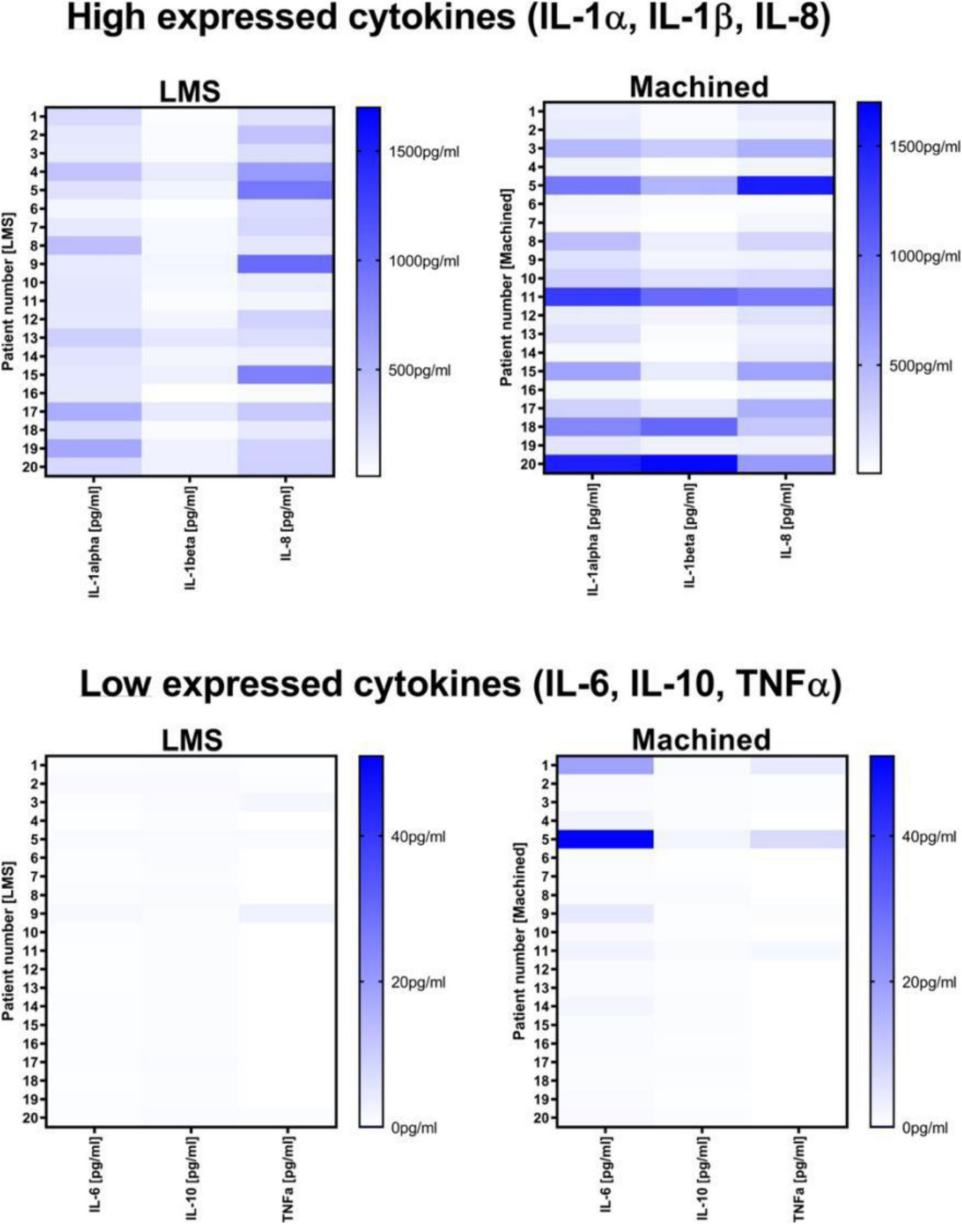
Cytokine analysis of IL-1α, IL-1β, IL-6, IL-8, IL-10, and TNF-α for LMS vs M abutments

IL-1β showed a significant correlation (*p*=0.0024) to radiologic bone loss (Fig. 3). The other variables IL-1α, IL-6, IL-8, and IL-10 had no significant correlation to radiological bone loss. The exploration of high expressed cytokines (IL-1α, IL-1β, IL-8) related to systemic and confounding factors revealed no abnormalities (Fig. 4).

## Discussion

The present study revealed no significant difference between LMS abutments and M abutments in the following clinical parameters recorded: width of keratinized mucosa (KM), width of mucosal recession (MR), plaque index (PI), clinical attachment level (CAL), and probing pocket depth (PPD). BOP and marginal alveolar bone loss were lower in the study group (LMS abutments) compared to the control group (machined abutments). Conform to the findings of bleeding on probing, the pro-inflammatory cytokine IL-6 was significantly higher and IL-α and IL-β were higher in the control group.

Due to the fact that inter-rater calibration was measured for PICF sampling and for probing force, BOP seems to be a critical diagnostic parameter indicating inflammatory peri-implant processes. This fact is supported by several studies. Ericsson and Lindhe observed in a dog study BOP in most of the healthy peri-implant sites [10]. In addition, multiple long-term clinical studies showed a poor correlation of BOP and peri-implant disease [32–34]. French et al. found out in a large cohort study that minimal BOP did not correlate with bone loss, in contrast to intense BOP and/or suppuration did [35]. Emecen-Huja et al. considered modest BOP in peri-implant soft tissue a state of subclinical chronic inflammation [36]. Thus, BOP assessment is reasonably to differ between peri-implant health and disease and could guide to over-diagnosis and over-treatment [37]. In addition, consequent diagnostic peri-implant probing could result in mechanical disruption and iatrogenic trauma to the peri-implant soft tissue similar to multiple abutment dis-/reconnection [38–40] and could affect negatively peri-implant tissue stability [41].

Implants treated with M abutments showed higher radiographic bone loss (*p*=0.016) indicating that LMS abutments may reduce peri-implant bone loss. These findings are in accordance with former studies confirming the hypothesis of fewer marginal bone loss in earlier clinical trials [42, 43]. Iglhaut et al. showed in a dog model that LMS abutments on LMS implants in a “one abutment one time” approach without disconnection resulted in the preservation of the marginal crestal bone levels compared to machined abutments [44]. Several clinical studies demonstrated lowered marginal bone loss in LMS implants and LMS abutments compared to LMS implants and M abutments [20–22]. Soft tissue attachment seems to be more likely in lower nanorough abutment surfaces [4, 5, 45]. In an in vivo trial by Geurs et al. demonstrated that laser microgrooved surface abutments showed a zone of epithelial attachment, and connective tissue integration throughout the machined surface abutments showed only epithelial attachment [46]. Inflammatory infiltrates following microbial colonization of the implant-abutment interface could stimulate epithelial downgrowth and could promote peri-implant bone loss [47]. Contamination of the abutment surface is reported to have a negative effect on the soft tissue integration on the implant surface [48]. By inhibiting epithelial downgrowth and better sealing against the highly contaminated oral cavity, laser microgrooved surfaces seem to promote a positive effect on marginal bone maintenance and might support peri-implant tissue health [44, 46, 48].

The hypothesis of the present study was that LMS abutments show lower signs of inflammation indicated by lower cytokine levels. The findings of our observational study support the hypothesis. Indeed, IL-6 and IL-10 levels were significantly different in the study cohort with LMS abutments in comparison with the M abutments group. Severino et al. reported significantly increased IL-6 in the crevicular fluid in peri-implantitis patients [49]. IL-6 acts as a pro-inflammatory cytokine in CD4+ T cell differentiation [50]. IL-6 as a pro-inflammatory cytokine was significantly lower in the LMS group. The reason might be actually the aforementioned tighter and more tooth-like orientation of collagenous fibers into the laser microgrooves of the LMS abutment, which could help prevent microbial offenses in the peri-implant sulcus and lead to less pro-inflammatory activity (lower IL-6 level) in implants treated with LMS abutments. In a clinical study by Schwarz and coworkers comparing the incidence of experimental peri-implant mucositis, no differences concerning BOP were found in M vs LMS abutments [14] although this method could be limited related to individual influence of probing forces [9, 10].

At the moment, there is a lack of knowledge about the immune response to well-tolerated titanium implants. Thomas et al. showed in an in vitro study that the pro-inflammatory mediators IL-1β, IL-6, and TNF-α might be a “normal” unspecific response to titanium particles and suggested that IL-10 might prevent from peri-implant inflammation by downregulating the pro-inflammatory cytokines IL-6 and TNF-α [31]. Accordingly, a second significant observation in our experiments was the difference between the control and study groups regarding IL-10. IL-10 levels were significantly higher in LMS abutments. This finding may support our hypothesis due to the anti-inflammatory effect of IL-10. The other investigated cytokines IL 1β and IL-1α were higher in the control group (M abutments) though not significantly. In a systematic review, Duarte et al. found moderate evidence for the association of peri-implantitis to increased pro-inflammatory cytokine levels in the peri-implant crevicular fluid. Evidence for the suitability use of selected cytokines as possible biomarkers for peri-implantitis is limited; thus, further studies are needed [17].

However, the present study has limitations. Due to the heterogeneity in implant systems, loading time (2.6 years vs 3.6 years) and the lack of a long-time evaluation the study results must be taken carefully. To date, there is no agreement and definition on normal cytokine levels in PICF, so a differentiation of physiological or pathological state is not possible. In the present study, IL-6 and IL-10 levels differ significantly, but it remains unclear whether the reported levels in both groups represent varying health statuses. Simultaneous collection of PICF around healthy adjacent teeth could be a prospective method to differentiate between peri-implant health and inflammation. Nevertheless, monitoring cytokine levels in PICF could be useful for the detection of clinical latent, early stages of peri-implant inflammation. To our knowledge, this was the first study comparing LMS abutments to standard machined titanium abutments in regard to peri-implant cytokine levels. Nevertheless, the concept of laser microgrooving surfaces is promising in regard to the peri-implant mucosal seal and the resulting hard tissue preservation but needs further investigation.

Within the limitations of this study, titanium implants provided with laser-microtextured surface abutments seem to show less pro-inflammatory and more anti-inflammatory activity and to have a positive impact on peri-implant crestal bone stability compared to machined titanium abutments.

## Conclusion

Within the limitations of this study, titanium implants provided with laser-microtextured surface abutments seem to demonstrate less pro-inflammatory and more anti-inflammatory activity and to show reduced radiographic bone loss compared to machined titanium abutments.

## Data Availability

The datasets used and analyzed during the current study are available from the corresponding author on reasonable request.

## Abbreviations

PICF: Peri-implant crevicular fluid
M: Machined surface
LMS: Laser-microtextured surface
PD: Pocket probing depth
FMPS: Full-mouth plaque index
BOP: Bleeding on probing
RBL: Radiographic bone loss
CAL: Clinical attachment level
MR: Mucosal recession
KM: Width of keratinized mucosa
IL-1α: Interleukin 1 alfa
IL-1β: Interleukin 1 beta
IL-6: Interleukin 6
IL-8: Interleukin 8
IL-10: Interleukin 10
